# Supplementary material for: EXPLORE: a novel deep learning-based analysis method for exploration behaviour in object recognition tests
Source: Sci Rep. 2023 Mar 14;13:4249. doi: 10.1038/s41598-023-31094-w (PMC10014875; doi:10.1038/s41598-023-31094-w)
Supplement: Supplementary file 1 — Supplementary Information 1. [file 41598_2023_31094_MOESM1_ESM.pdf]

Supporting information

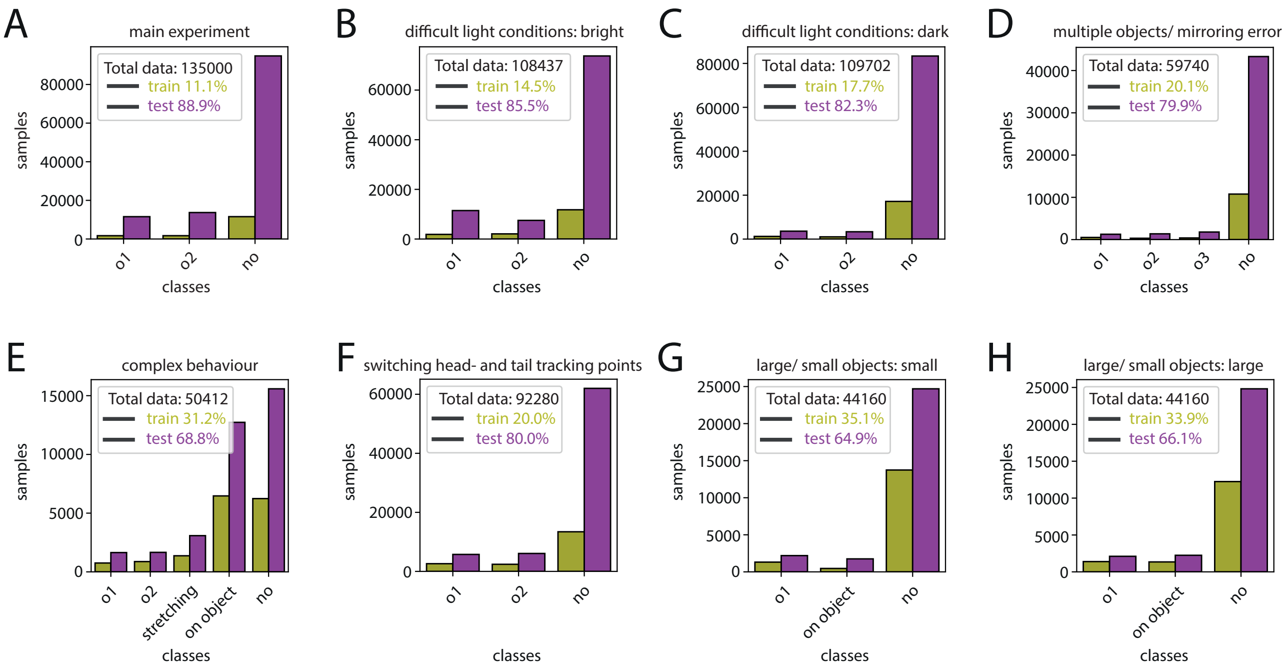

Supplementary Fig. 1. Distributions of different data sets used to evaluate EXPLORE (A)-(H) The x-axis shows the different classes used for the experiments split by training- and test data and the y-axis shows the amount of samples used respectively.

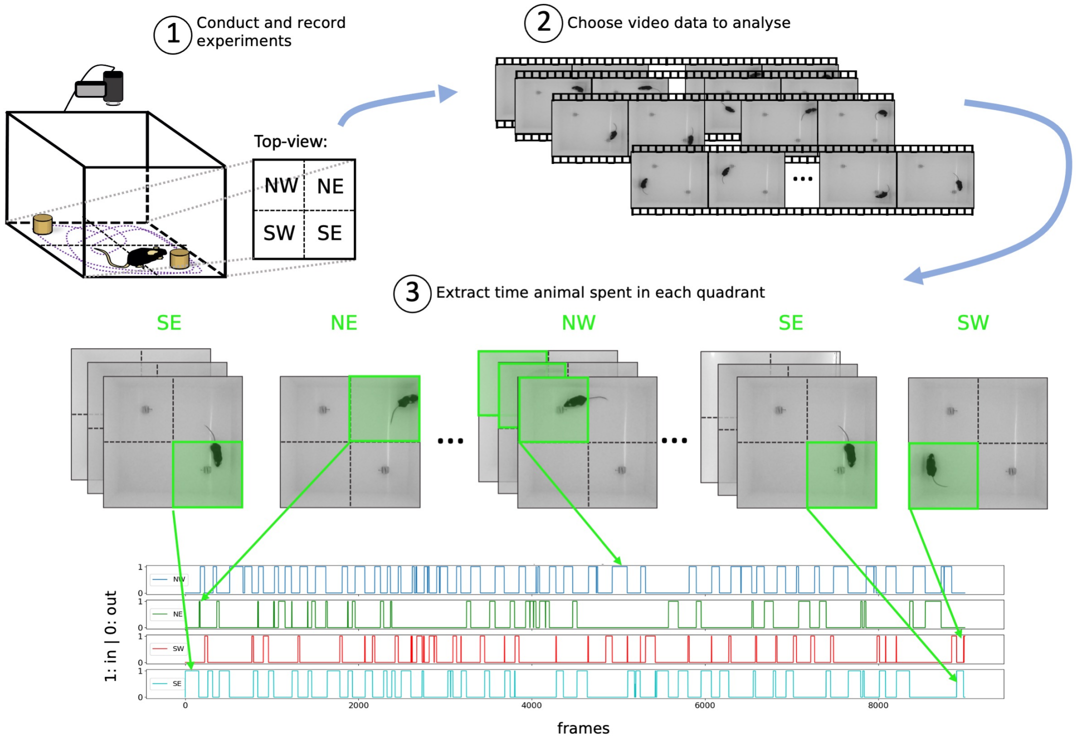

Supplementary Fig. 2. Overview of the quadrant exploration analysis. **(1)** Conduct and record experiments. **(2)** The videos to analyse can be selected. **(3)** In each frame, the mean pixel intensity for each quadrant is calculated. Then a threshold function is used to convert the values for each quadrant to "0" or "1" (the quadrant with the animal is assigned the value "1").

I Quadrant analysis: Testing on empty arena for quadrant preference of the animals

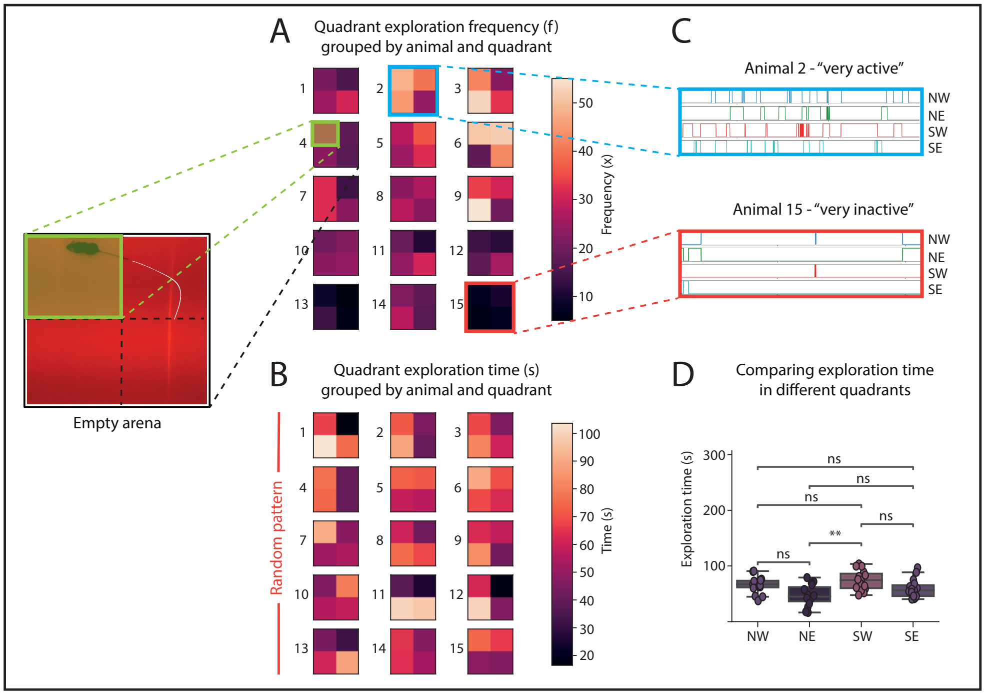

II Quadrant analysis: Testing on movement behavior in an arena filled with objects

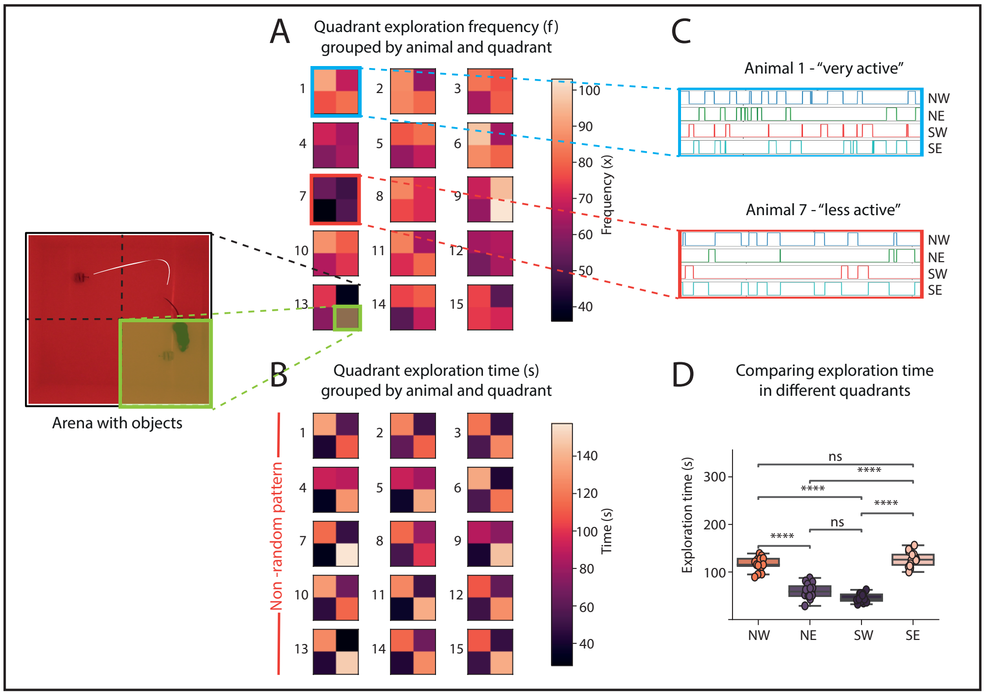

Supplementary Fig. 3. Quadrant exploration analysis can be used to qualitatively and quantitatively assess rodent behaviour and abundance in the quadrants of an experiment arena. (I) Running the quadrant exploration analysis on a set of experiment videos, where no object is in the arena. **A,B:** Heatmaps for the 15 assessed videos: Each numbered square represents the summed exploration frequency (**A**) or the summed exploration time (**B**) per animal with the four quadrants of the experiment arena (north-west (NW), north-east (NE), south-east (SE), south-west (SW)). **C:** two examples (2 animals) of the frequency plots for each of the quadrants: Animal 2 shows a lot of activity overall, animal 15 not much. **D:** To assess if the animals spent equal time in each quadrant and had no preference, the total exploration time for each quadrant can be compared: We used a paired Wilcoxon test with bonferroni post-hoc and found no significant difference between the quadrants. In **B** it can also qualitatively be seen that there is no "pattern" visible: Animals seem to have randomly visited the quadrants. **(II)** As a placeholder for general cues that could potentially bias the experiment, we run the quadrant exploration analysis on a set of experiment videos, where 2 objects are located NW and SE in the arena. **A,B,C:** same as in **I**. **D:** Here, it can be seen that the summed exploration times for each quadrants are significantly different between NW and NE, NW and SW, NE and SE, SW and SE. **B** also shows that there is a clear "pattern" visible: Animals explored NW and SE (object locations) longer than NE and SW. Asterisks showing significance level: ns ≥ 0.05, * ≤ 0.05, ** ≤ 0.01, *** ≤ 0.001, **** ≤ 0.0001.

Supplementary Video 1. Example video of EXPLOREs performance on our main data set. EXPLORE correctly predicts when the animal is exploring the two objects (Whenever EXPLORE counts exploration behavior, the objects are marked with a coloured frame).

Supplementary Video 2. EXPLORE video example on data set "difficult light conditions: bright". While keypoint tracking software fails, EXPLORE is able to classify the behaviours as exploration (Whenever EXPLORE counts exploration behavior, the objects are marked with a coloured frame).

Supplementary Video 3. EXPLORE video example on data set "difficult light conditions: dark". As in Supplementary video 2, EXPLORE sufficiently detects exploration behavior, even under these difficult light conditions (Whenever EXPLORE counts exploration behavior, the objects are marked with a coloured frame).

Supplementary Video 4. Sample video of EXPLORE performing on multiple objects EXPLORE is able to correctly classify behaviour on multiple objects. Whenever EXPLORE counts exploration behavior, the objects are marked with a coloured frame.

Supplementary Video 5. A video sample revealing a more versatile usage of EXPLORE. EXPLORE is able to precisely capture different complex features of behaviour, defined by the user (Whenever EXPLORE counts exploration behavior, the objects are marked with a coloured frame, behaviour are marked with small coloured frames).

Supplementary Video 6. Video sample demonstrating the performance of EXPLORE, where keypoint tracking software would lose or switch tracking points. EXPLORE correctly predicts when the animal is exploring the two objects (Whenever EXPLORE counts exploration behavior, the objects are marked with a coloured frame).

Supplementary Video 7. Video sample depicting the prediction of exploration behavior by EXPLORE when using small objects. EXPLORE captures what the user defines as exploration behavior independent of the size of the object, leading to correct labels (Whenever EXPLORE counts exploration behavior, the object is marked with a coloured frame).

Supplementary Video 8. Video sample depicting the prediction of exploration behavior by EXPLORE when using large objects. EXPLORE captures what the user defines as exploration behavior independent of the size of the object, leading to correct labels (Whenever EXPLORE counts exploration behavior, the object is marked with a coloured frame).
